# The Prevalence of Dental Fear and Its Relationship to Dental Caries and Gingival Diseases Among School Children in Wardha

**DOI:** 10.7759/cureus.46360

**Published:** 2023-10-02

**Authors:** Shivangi Agarwal, Manoj Chandak, Amit Reche, Prachi V Singh

**Affiliations:** 1 Department of Public Health Dentistry, Sharad Pawar Dental College and Hospital, Datta Meghe Institute of Higher Education and Research, Wardha, IND; 2 Department of Conservative Dentistry and Endodontics, Sharad Pawar Dental College and Hospital, Datta Meghe Institute of Higher Education and Research, Wardha, IND

**Keywords:** dental treatment, gingival condition, dental caries, dental anxiety, children

## Abstract

Background

Children who are afraid of the dentist have terrible behavioral effects, and one of those effects is that they have a preconceived concept that getting treatment would be unpleasant. Such fear and anxiety can lead to avoidance of dental care. These patients must be recognized and their concerns addressed as soon as possible. It is, therefore, important to highlight the connection between the constructs that target the development of dental fear and anxiety, including its outcome in children concerning the prevalence of dental diseases.

Aims and objectives

To assess the prevalence of dental anxiety and its correlation to dental caries and gingivitis in students in Wardha aged six to 12 years old.

Methods

Two hundred schoolchildren between the ages of six and 12 were chosen at random, with 100 boys and 100 girls. Children had an oral examination utilizing the decayed, missing, and filled teeth (DMFT) Index as well as the Loe and Silness gingival index (GI), as well as a modified version of the dental fear survey questionnaire.

Results

In the study population, the prevalence of low to moderate "general dental fear" was 47%, whereas the frequency of high dental fear was 14%. The mean DMFT (1.80 ± 1.76) and GI (1.04 ± 0.52) of boys did not differ substantially from the DMFT (1.94 ± 81.02) and GI (0.97 ± 0.53) of girls (P > 0.05).

Conclusion

In terms of DMFT and gingival scores, there was not any obvious distinction between male and female children. Additionally, there was no connection found between "general dental fear" and either the DMFT or GI scores. Dental fear scores decreased with increasing age.

## Introduction

Dental anxiety denotes a sense or response to a recognized potential threat, whereas dental fear is a distinct sort of phobia that affects an individual's emotional response to a terrifying stimulus. This is something that the subconscious brain knows about [1]. Children have traditionally had a negative perception of dental care, which has instilled fear and worry in them. Such dental anxiety has a detrimental influence on their behavior, preventing them from adapting to the clinical scenario and preventing them from seeking prompt dental therapy. According to Beena et al., the fourth most prevalent phobia is the fear of visiting the dentist [2]. Many nations have identified dental fear in kids as a public health issue [3,4]. This issue creates a challenge for both patients and dentists since it may result in neglecting dental care [4,5]. According to research, the impacts of a child's dental concerns may potentially last into adolescence and may result in the avoidance of dental care or disruptive conduct while receiving treatment [6]. Because of this, it is crucial that the dental health expert spot kids who have dental anxiety and use the best pediatric care strategies at the youngest age feasible [7,8].

Oral health, along with access to health, nutrition, cancer, HIV, and heart disease, has been named as one of the 10 key health indicators. Speaking, grinning, smelling, and eating are just a few of the basic human functions that are made possible by good dental health. It's also crucial for relationships with others, getting along in society, and being successful financially. In addition to causing expensive, painful, and disabled health concerns, poor oral health has major repercussions. Therefore, to avoid these consequences it is important to avail early and accurate dental treatment. Truth be told, the condition of your oral cavity has a huge impact on your overall well-being, the expense of your medical care, and your quality of life. People frequently neglect and undervalue the importance of the oral cavity in terms of our health. An increasing amount of studies have found a link between dental health and significant problems impacting millions of individuals, including diabetes, Alzheimer's disease, and other chronic diseases. The goal is to close the "gap" that has historically existed between the medical and dental professions and to use these findings to lower the risk of such chronic diseases.

Milgrom et al. proposed that conditioning is a significant underlying cause of dental anxiety in children as well as adolescents [9]. Unfavorable dental experiences as a youngster, negative mindsets in the family, and terrible experiences from past dental operations are all potential reasons for dental anxiety. All symptoms of the said condition are now collectively referred to as "dental fear and anxiety" (DFA), and the terms are equivalent [1]. DFA has been discovered to differ in substance and structure across various communities and populations. According to several studies, the occurrence of dental fear in various populations ranges from 4 percent to 43 percent [10]. Children having higher active caries lesions are more afraid than most others, most likely as a result of a past bad dental experience. Fear of dental treatments might prevent patients from complying with their treatment plans leading to greater plaque accumulation and bleeding gums and thus, worsening their gingival health. This may be because these dentally worried individuals were less likely to embrace preventative dental habits, necessitating significant dental care. It suggests that dental fear affects access to appropriate preventative and treatment services, leading to oral health deterioration [11]. The cause may be because these dentally nervous individuals were less inclined to embrace preventative dental habits, necessitating increased comprehensive dental care. This revealed that dental anxiety affects the appropriate preventative and therapeutic options, causing oral health to deteriorate [12].

There have been studies published in the past that establish a correlation between the prevalence of dental fear and dental diseases. However, most of these studies are either conducted on adolescents or among older age group people [3-5]. Also, relatively few studies have examined the implications for gingival health [4]. Therefore, the current study is carried out to determine the prevalence of dental fear and its relationship to dental caries and gingival diseases among schoolchildren aged six to 12 in Wardha City. This research has linked schoolchildren's level of dental anxiety, and how important it is for developing suitable approaches for improving and promoting oral health among youngsters in the Wardha population. As a result, the cross-sectional study is created and carried out to examine and consider the mentioned objectives:

1. To determine the level of dental anxiety among school children aged six to 12.

2. To characterize the sex distribution of dental anxiety amongst schoolchildren based on the origins of their anxiety.

3. To evaluate dental status in boys and girls about decaying, missing, and filled teeth (DMFT) and gingival index (GI).

4. To determine the occurrence of dental caries as well as gingivitis, as well as correlate it with the impact of dental fear.

## Materials and methods

Study design, setting, and participants

Data for this investigation were obtained prospectively from children going to different schools in Wardha, Maharashtra aged between six and 12 years October 2022 and January 2023. There is little research on dental fear among this age group of schoolchildren in India, hence this particular age group was chosen. The simple random method was used to select three private English medium schools that were present in the rural region of the Wardha district. The study comprised participants residing within a 40-kilometer radius of Wardha district and the study area was a rural area. It was ensured that a teacher was present to brief the children throughout the process which took place under the observation of a pedodontist.

Ethics consideration and sample size

This was a cross-sectional research, and the Institutional Ethical Committee of Datta Meghe Institute of Medical Sciences (DU), Wardha gave its approval to the necessary methodology, DMIMS(DU)/IEC/2022/1191. Two hundred school students (100 boys along with 100 girls) aged between six and 12 years were selected by simple random method. Written informed consent was obtained from the parents of all the students along with the acceptance to the examination and the study participants were well informed about the study. Consent was also obtained from the school authority for conducting the examination.

Study criteria (inclusion and exclusion)

Inclusion Criteria

Children between the ages of six and 12 who had never received dental treatment previously, however, have visited a dental facility with someone else, and as a result, they are well-versed in all dental procedures.

Exclusion Criteria

Children with systemic diseases, mental problems, or any physical impairment.

Data collection

Questionnaire

The children were asked to fill out an updated version of the dental fear survey questionnaire. The dental fear scale (DFS) of Klienknecht et al. was modified and implemented comprising a 10-item questionnaire. Children were requested to rate their level of dental fear on a scale of one to four: not terrified-1, a little terrified-2, somewhat terrified-3, and immensely terrified-4. As a result, overall scores varied from 10 to 40. The following were the 10 dental treatment-related questions: Watching the dentist walk in, sitting on the dental chair, watching the dentist walkout, during a dental checkup, viewing the syringe needle, experiencing the syringe needle inserted, witnessing the drill, hearing the drill, during their treatment and experiencing discomfort thereafter anesthesia administered locally. Individuals who categorized themselves as very or extremely terrified were classified as high fear, while those who said they were not scared or somewhat scared were classified as low fear. The teacher handed out the questionnaire to the students following a brief explanation in the classroom under the observation of the pedodontist. Children were not allowed to discuss among themselves; instead, a translator would translate their doubts from English into their local language and back again. The survey approximately took 15 minutes to complete.

Clinical Examination

Following the completion of the questionnaire, a qualified examiner performed a dental examination in the school setting. By placing the child on a chair next to a window and using natural light for a visual examination, dental caries in children were examined. The WHO-developed diagnostic standards for caries were employed [13]. Only permanent teeth were used to record DMFT values while decayed, extracted-filled teeth were recorded in deciduous teeth. Using the Loe and Silness GI [14], gingival health was also evaluated by examining the gingiva surrounding the six indexed teeth. The questionnaires were obtained as well as collected for data analysis following the oral examination.

Data analysis

Statistical analysis was done by Statistical Package for the Social Sciences (IBM SPSS Statistics for Windows, IBM Corp., Version 23.0, Armonk, NY). Dental fear, DMFT, and GI underwent descriptive analysis, with the mean and standard deviations calculated. The correlation of demographic characteristics with the questionnaire was determined using Chi-squared statistics. The relationship between dental fear and dental caries or gingival diseases was examined using Pearson's correlation test. P<0.05 was regarded as statistically significant for all results.

## Results

Two hundred schoolchildren in all, aged six to 12, were interviewed and examined (100 males and 100 females). The age and gender distribution of the research population is shown in Table 1. The children's average age was determined to be 9.04, with a standard deviation of 2.009 which was almost identical for boys and girls. Depending on their level of "general dental fear," the distribution by sex is shown in Table 2. It was shown that 4.5% of males and 13% of females had severe dental fear, compared to 67.9% of males and 79% of females who had moderate dental fear, and 27.6% of males and 8% of females who had mild dental fear. Girls were considerably more inclined than boys to indicate general dental fear (P < 0.001).

**Table 1:** Age and gender distribution of the research population (n=200)

Variables	Male	Female
Sex	100	100
Mean Age	9.04±2.009	9.04±2.009

**Table 2:** Gender distribution according to “general dental fear”

General Dental Fear	Male (%)	Female (%)	P value
Mild Dental Fear	27.6%	8%	P <0.001
Moderate Dental Fear	67.9%	79%	
Severe Dental Fear	4.5%	13%	

**Figure 1:**
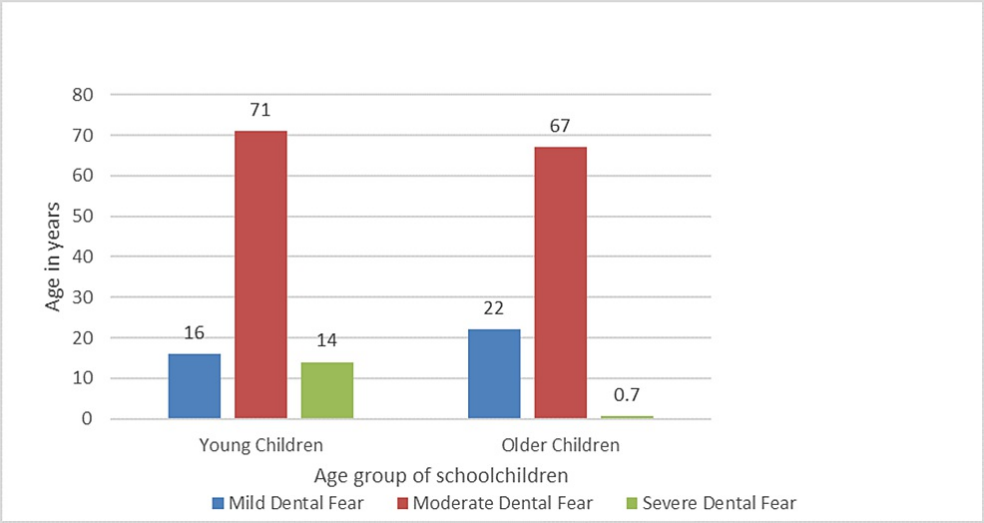
Distribution of the age group of school children by general dental fear

Figure 1 displays the distribution of schoolchildren's age groups by general dental fear. It was shown that young children between the ages of six and nine showed 14% severe dental fear and 71% moderate dental fear. Children aged between 10 and 12 showed a 67% moderate fear of the dentist and a 0.7% severe fear.

**Figure 2:**
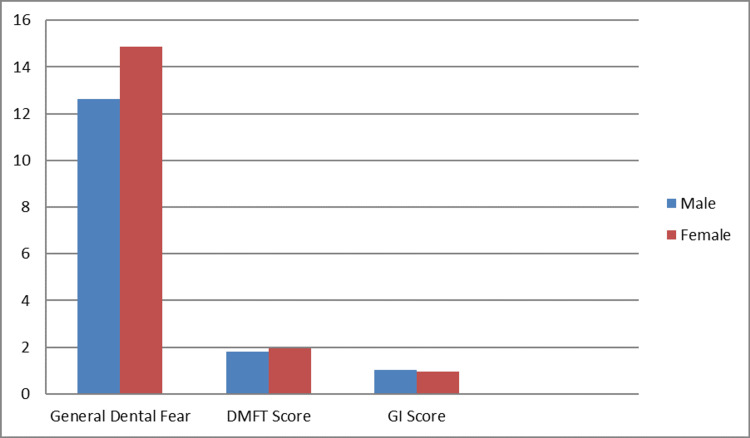
Mean values of Dental Fear Score related to the mean of DMFT and GI scores DMFT: decayed, missing, and filled teeth, GI: gingival index

Table 3 compares the mean and standard deviation of the DFS scores with those of the DMFT and GI. The mean general dental fear score for boys (12.62 ± 3.46) was seen to be slightly lesser than that of the girls (14.86 ± 3.97). The mean DMFT score for boys (1.80 ± 1.76) was not substantially different from that of the girls (1.94 ± 1.83) (P = 0.45). Boys' mean GI scores (1.04 ± 0.52) like girls' (0.94 ± 0.57) did not differ greatly from one other (P = 0.180). Figure 2 shows the distribution of mean DFS scores in males and females and compares it with that of the DMFT and GI scores.

**Table 3:** Mean and SD of dental fear score related to the mean ± SD of DMFT and GI SD: standard deviation, DMFT: decayed, missing, and filled teeth, GI: gingival index

Variables	Male (n=100)	Female (n=100)	t value	P value
General Dental Fear	12.62 ± 3.46	14.86 ± 3.97	5.878	0.005
DMFT Score	1.80 ± 1.76	1.94 ± 1.83	0.758	0.45
GI Score	1.04 ± 0.52	0.94 ± 0.57	1.345	0.180

## Discussion

Pramila M et al. conducted a study on 1452 children from the 12-15 years age group in Bangalore study. By using a straightforward random procedure, eight government high schools were chosen from Bangalore city's two zones. All the children who took part in this study were given the single-item Dental Anxiety Question to gauge their level of dental anxiety. They also had their mouths checked for dental caries and gingivitis using the Community Periodontal Index (CPI) and DMFT indices, respectively. The results were significant concerning the current study. The research population's prevalence of severe dental anxiety was 23.4%. High fear was substantially correlated with the mean MT. Despite the large number of research examining the link between dental fear and the likelihood of developing caries, relatively few studies have examined the implications for gingival health [4,11]. High dental fear and the presence of blood on light probing were shown to be significantly correlated.

DQ Taani et al. conducted research to assess the link between these characteristics and the levels of dental anxiety, dental caries, and gingivitis among 12- to 15-year-old pupils in northern Jordan's Irbid Governorate, research was conducted. A straightforward random process was used to choose two schools from each of the governorates of Irbid's five geographical regions. All the students (1021) from the 10 schools chosen for this study filled out a modified version of Kleinknecht's DFS questionnaire. Using the DMFT Index and the Loe and Silness GI, children had oral checkups to examine dental caries and gingival health. According to the research's findings, 43% of the people in the research showed low to moderate levels of "general dental fear," however 10% showed extremely elevated levels. In correlation with the current study, girls were more likely than boys to report having a general fear of getting dental work done. Boys' mean DMFT (2.89) and GI (1.80) did not differ substantially (P >0.05) from girls' mean DMFT (3.37) and GI (1.53) [4].

NK Chellappah et al. conducted research to evaluate dental anxiety among 12- to 14-year-old primary school-going children in Singapore. Sixty-six children were identified as having significant fear, yielding an overall prevalence rate of 177 anxious children per 1000 population after adjusting for gender and race. Girls exhibited 2.64 times greater fear than boys. The results were significant concerning the current study. In terms of prevalence rates, there were no observable racial disparities. Children who report having dental anxiety are nearly three times more likely to have high-state anxiety than low-state anxiety children. The study also concluded that dental fear has several intervening factors, one of which is accessibility to dental treatment [3].

GS Prathima, Abhishek Shaji Varghese, et al. performed research to assess the frequency of dental anxiety among children aged six to 12 attending Puducherry schools, as well as its associations with dental caries and gingival disease. Four hundred schoolchildren between the ages of six and 12 were chosen at random, 200 males and 200 girls. Children had an oral examination utilizing the DMFT index and the Loe and Silness GI, as well as a modified version of the DFS questionnaire. In correlation to the current study, the prevalence of "general dental fear" ranged from mild to moderate (46%), to severe (72%). In terms of DMFT and gingival scores, there were no discernible differences among young boys and girls. Age-related declines in dental fear ratings were seen. Dental fear did not correlate with DMFT or GI ratings.

According to Kleinknecht RA et al., procedures involving the drill and needle were the most often dreaded parts of dental care [15]. Children in the current study were extremely terrified of "injections" and "dentist drills." According to research by Milgrom et al. on children aged five to 11 years, more than 66% of children develop their fears at this time [9]. In the present study, it was found that compared to older kids, young kids aged six to nine had 71% moderate dental fear and 14% severe dental fear. According to Bedi et al. [5], high dental anxiety was linked to females' anxiety levels being greater than males', which was consistent with the current findings. Males are more likely to communicate their anxiety through other emotions like anger or frustration [16]. As children become older, LeBaron and Zeltzer [17] speculate that they may learn to manage how they exhibit their fear, which is consistent with the current study's finding that dental fear ratings declined with age. A reliable indicator of dental caries, according to Kruger et al.'s study, is dental fear itself, which may also be a risk factor for the development of dental caries [18]. The DMFT and gingival values, as well as "general dental fear," were not significantly different in the present investigation. According to Schuller et al., there is no statistically significant difference in the DMFT scores between those with high and low dental fear, even though the proportion of missing teeth among people with high dental fear is over 50% greater than that of people with low fear [19]. This was taken to mean that patients with a high level of dental fear preferred to have their teeth extracted over-restored. These people are a perfect fit for the hypothesized "vicious cycle" of fear, in which people with dental anxiety avoid getting the care they need, which worsens their issues and a greater propensity for further dental appointments to be made as a result of symptoms or emergencies, which are almost always more intrusive and unpleasant. In his research, SA Alamri et al. revealed that DFS, which was employed in this study, exhibits strong internal consistency and reliability, as a result, it may be used to more precisely quantify dental anxiety among various populations [20].

Pediatric dentists face a difficult problem when it comes to diagnosing, managing behavior, and treating patients who have dental anxiety. Every attempt should be taken to find out if the child is afraid of the dentist before performing any dental procedure. As a result, it will be possible to handle each kid differently depending on the things that make them fearful and to utilize the required behavior modification strategies to ensure that the dental process is comfortable as well as uneventful. The application of epidemiologic concepts such as caries activity testing, fluoride administration, and timely efforts to utilize preventative fissure sealants must be done with great caution. To prevent the kid from feeling pain and reduce the need for early-onset injections, repeated oral health checks, oral hygiene instruction, and parental counseling were necessary to lower the degree of dental fear in children [3].

Study limitations

Although significant connections were discovered between children's dental fear and oral health, the current study only partially addresses the problem of dental fear. The study employed one specific measure of dental fear, without any psychometric assessment of fear conducted. Despite the questionnaire's reliability and validity having been established, it may generalize the different features of dental fear and anxiety, unlike the other multi-item measures such as Corah’s Dental Anxiety Scale (DAS), the Modified Dental Anxiety Scale, and the Dental Fear Survey Schedule [10]. Nevertheless, the findings shown here allow us to consider a solution. Another limitation is that the cross-sectional design of the current study means that it can solely provide information on connections, not causality. A longitudinal cohort study must be created to prove causation [15].

## Conclusions

The results of the current investigation revealed that gender was a major predictor of recorded dental fear, with girls reporting dental fear at a higher rate than boys. It was also seen that with advancing age, dental fear scores were also reduced. In terms of DMFT and gingival scores, there was not any obvious distinction between male and female children. Additionally, there was no connection found between "general dental fear" and either the DMFT or GI scores. The absence of a link in the present research might be explained by the fact that responses to various dental treatment cues vary greatly among dentally worried people. Therefore, by addressing dental fear, and desensitizing them with positive reinforcements we may enhance their dental attendance as well as their oral health, particularly for individuals who experience severe dental anxiety. So, we must ensure to make the child's dental treatment pleasant while also instilling a favorable attitude toward future dental operations.

## References

[1] Rubin JG, Slovin M, Krochak M (1988). The psychodynamics of dental anxiety and dental phobia. Dent Clin N Am.

[2] Beena JP (2013). Dental subscale of children's fear survey schedule and dental caries prevalence. Eur J Dent.

[3] Chellappah NK, Vignehsa H, Milgrom P, Lam LG (1990). Prevalence of dental anxiety and fear in children in Singapore. Community Dent Oral Epidemiol.

[4] Taani DQ, El-Qaderi SS, Abu Alhaija ES (2005). Dental anxiety in children and its relationship to dental caries and gingival condition. Int J Dent Hyg.

[5] Bedi R, Sutcliffe P, Donnan PT, McConnachie J (1992). The prevalence of dental anxiety in a group of 13- and 14-year-old Scottish children. Int J Paediatr Dent.

[6] Klaassen MA, Veerkamp JSJ, Aartman IHA, Hoogstraten J (2002). Stressful situations for toddlers: indications for dental anxiety?. J Dent Child.

[7] Yamada MK, Tanabe Y, Sano T, Noda T (2002). Cooperation during dental treatment: the Children's Fear Survey Schedule in Japanese children. Int J Paediatr Dent.

[8] Holmes RD, Girdler NM (2005). A study to assess the validity of clinical judgement in determining paediatric dental anxiety and related outcomes of management. Int J Paediatr Dent.

[9] Milgrom P, Mancl L, King B, Weinstein P (1995). Origins of childhood dental fear. Behav Res Ther.

[10] Armfield JM, Spencer AJ, Stewart JF (2006). Dental fear in Australia: who's afraid of the dentist?. Aust Dent J.

[11] Pramila M, Murthy AK, Chandrakala B, Ranganath S (2010). Dental fear in children and its relation to dental caries and gingival condition-a cross sectional study in Bangalore city, India. Int J Clin Dent Sci.

[12] Esa R, Ong AL, Humphris G, Freeman R (2014). The relationship of dental caries and dental fear in Malaysian adolescents: a latent variable approach. BMC Oral Health.

[13] Radić M, Benjak T, Vukres VD, Rotim Ž, Zore IF (2015). Presentation of DMFT index in Croatia and Europe. Acta Stomatol Croat.

[14] Löe H (1967). The gingival index, the plaque index and the retention index systems. J Periodontol.

[15] Kleinknecht RA, Klepac RK, Alexander LD (1973). Origins and characteristics of fear of dentistry. J Am Dent Assoc.

[16] Gadbury-Amyot CC, Williams KB (2000). Dental hygiene fear: gender and age differences. J Contemp Dent Pract.

[17] LeBaron S, Zeltzer L (1984). Assessment of acute pain and anxiety in children and adolescents by self-reports, observer reports, and a behavior checklist. J Consult Clin Psychol.

[18] Kruger E, Thomson WM, Poulton R, Davies S, Brown RH, Silva PA (1998). Dental caries and changes in dental anxiety in late adolescence. Community Dent Oral Epidemiol.

[19] Schuller AA, Willumsen T, Holst D (2003). Are there differences in oral health and oral health behavior between individuals with high and low dental fear?. Community Dent Oral Epidemiol.

[20] Alamri SA, Alshammari SA, Baseer MA, Assery MK, Ingle NA (2019). Validation of Arabic version of the Modified Dental Anxiety Scale (MDAs) and Kleinknecht’s dental fear survey scale (DFS) and combined self-modified version of this two scales as dental fear anxiety Saale (DFAS) among 12 to 15 year Saudi school students in Riyadh City. J Int Soc Prev Community Dent.

